# Supporting carers: health care professionals in need of system improvements and education - a qualitative study

**DOI:** 10.1186/s12904-019-0444-3

**Published:** 2019-07-16

**Authors:** Ingebrigt Røen, Hans Stifoss-Hanssen, Gunn Grande, Stein Kaasa, Kari Sand, Anne Kari Knudsen

**Affiliations:** 1 European Palliative Care Research Centre (PRC), Department of Clinical and Molecular Medicine, Norwegian University of Science and Technology (NTNU), 4. etg. Kunnskapssenteret vest, St. Olavs hospital, 7006 Trondheim, Norway; 2 St. Olavs hospital HF, Trondheim University Hospital, 4. etg. Kunnskapssenteret vest, St. Olavs hospital, 7006 Trondheim, Norway; 3 Center of diakonia and professional practice, VID Specialized University, Oslo, Norway; 4 Division of Nursing, Midwifery and Social Work, The University of Manchester, Manchester, England; 5 European Palliative Care Research Centre (PRC), Department of Oncology, Oslo University Hospital and Institute of Clinical Medicine, University of Oslo, Oslo, Norway; 6 Department of Oncology, Oslo University Hospital, Oslo, Norway; 7 University of Oslo, Oslo, Norway

**Keywords:** Health care professionals, Oncology, Palliative care, Family carers, Carer support, Needs assessment, Integration

## Abstract

**Background:**

Health care professionals should prevent and relieve suffering in carers of patients with advanced cancer. Despite known positive effects of systematic carer support, carers still do not receive sufficient support. Carers have reported to be less satisfied with coordination of care and involvement of the family in treatment and care decisions than patients. In a rural district of Mid-Norway, cancer palliative care services across specialist and community care were developed. Participants’ experiences and opinions were investigated as part of this development process.

**Methods:**

The aim of this qualitative study was to explore and describe health care professionals’ experiences with carer support from their own perspective. Data were collected in focus groups. Purposeful sampling guided the inclusion. Six groups were formed with 21 professionals. The discussions were audio-recorded, transcribed, and analyzed using systematic text condensation.

**Results:**

In the analyzis of the focus group discussions, ten categories emerged from the exploration of health care professionals’ carer support, assessment of needs, and factors hampering carer support: 1) dependent on profession, role, and context, 2) personal relationship, 3) personal skills and competence, 4) adjusted to the stage of the disease, 5) informal assessment of carers’ needs, 6) lack of education 7) lack of systems for carer consultations, 8) lack of systems for documentation, 9) lack of systems for involving GPs, and 10) lack of systematic spiritual care.

**Conclusions:**

Health care professionals built a personal relationship with the carers as early as possible, to facilitate carer support throughout the disease trajectory. Systematic carer support was hampered by lack of education and system insufficiencies. Organizational changes were needed, including 1) education in carer support, communication, and spiritual care, 2) use of standardized care pathways, including systematic carer needs assessment, 3) systematic involvement of general practitioners, and 4) a system for documentation of clinical work with carers.

## Background

The National Institute for Health and Care Excellence in UK (NICE) defines carers as follows: “Carers, who may or may not be family members, are lay people in a close supportive role who share in the illness experience of the patient and who undertake vital care work and emotion management” [1]. The World Health Organization’s (WHO) definition of palliative care states that health care professionals (HCPs) should prevent and relieve suffering in carers. Early identification of carers’ physical, psychological, social, and spiritual pain and needs, as well as support to enhance carers’ coping during caring, and in bereavement, has been recommended [2]. Guidelines for HCPs’ carer support endorsed by international organizations included needs assessment, development of a plan for carer support, and preparing carers for the patient’s death [3]. In Norway, there were specific guidelines addressing these aspects for carer support in general [4], and for carers in cancer palliative care in particular [5].

Integration of oncology and palliative care has in randomized controlled trials (RCTs) shown to improve carers’ satisfaction with care, levels of depression, and stress burden [6–8]. A meta-analysis of 29 randomized clinical trials analyzed interventions to support carers of cancer patients. The interventions delivered psychoeducation, skills training, and therapeutic counselling, and significantly lowered carer burden, and improved coping and quality of life [9]. Despite positive effects of systematic carer support, carers still do not receive sufficient support [9]. Carers of patients with advanced cancer have reported to be less satisfied with coordination of care and involvement of the family in treatment and care decisions than the patients [10]. Carers reported that HCPs give limited information about available carer support, and that carers’ support needs are only occasionally addressed in discussions between nurses, patients, and carers [11]. Studies have reported that HCPs misjudge what carers need [12, 13]. A recent review including 50 RCTs investigating interventions to support carers, found that the majority of interventions demanded more than 3 h, usually performed by research staff. The authors concluded that there was a need for models of carer support being feasible in clinical practice, and recommended use of existing tools for assessment of carers’ needs [14]. One example of a short and feasible tool for assessment of carers’ needs is the Carer Support Needs Assessment Tool (CSNAT) [15]. Assessing carers’ needs was recommended in the recent Lancet Oncology Commision on cancer palliative care [7]. CSNAT was recommended to achieve more systematic carer support in a Norwegian governmental report on palliative care [16].

In Norway, health care services are mainly public, and oncology and palliative care are offered as an integrated part of the health care system. General practitioners (GPs), home care services, and nursing homes constitute community care. In specialist care, there are several local hospitals at secondary care level collaborating closely with community care, and six university hospitals offering tertiary care [17]. The Norwegian cancer strategy [18] underlined the need for integration between oncology and palliative care, and recommended the use of standardized care pathways to improve quality and coordination of care both for patients and carers, as also recommended in the recent Lancet Oncology Commission publication [7]. The present study was performed in the context of a health care services development project in Orkdal, a rural region in Mid-Norway, aiming at improving cancer care through integration of oncology and palliative care services, and through improved coordination of care across specialist and community care [19, 20]. ln 2012, a combined oncology and palliative care outpatient clinic (hereafter Integrated Clinic) was established at the local hospital, being organized as part of the Cancer Clinic at Trondheim University Hospital. A collaborative project between specialist and community care in 13 municipalities (about 56 000 inhabitants) was established with formal agreements, and evaluated in a prospective, still ongoing study, including both cancer patients, their carers, and HCPs [19]. From 2013 a complex intervention was developed, consisting of a standardized care pathway (SCP), an educational program, and an information strategy [20]. Qualitative studies to assess experiences of involved patients, carers, and HCPs were planned as part of the development process of the cancer palliative care services in the Orkdal region; the present study was one of these.

## Methods

### Aims of the study

This study was performed to explore and describe health care professionals’ (HCPs) carer support within cancer palliative care in a rural district in Mid-Norway (Orkdal), addressing the following research questions:

1. How do health care professionals in the Orkdal region support carers of patients with advanced cancer?
2. How do health care professionals in the Orkdal region assess the needs of carers of patients with advanced cancer?
3. What hampers the support to carers of patients with advanced cancer provided by health care professionals in the Orkdal region?

### Design

A qualitative methodology using semi-structured focus group interviews with health care professionals (HCPs) working with carers of patients with advanced cancer was chosen for exploring HCPs’ experiences [21, 22]. During focus groups, qualitative information about a subject is collected through group discussions [23, 24]. The method may bring up themes that the researcher did not anticipate, and produce concrete stories and in-depth discussions through the group interaction [22, 23].

### Participants and setting

Four focus groups with four-five participants in each group were planned, or until saturation. A purposeful sampling approach [25] was used for the inclusion of HCPs, aiming at achieving variation in profession and healthcare setting (specialist/community care). About 1360 HCPs (number fluctuating) were working in the region in the recruitment period. Among these, oncologists, general practitioners (GPs), cancer nurses, nurses, and assistant nurses with more than 1 year of working experience in a permanent position within cancer palliative care in the Orkdal region (at the palliative care unit of Trondheim University Hospital, at Orkdal Hospital, at GPs’ office, in nursing homes, or in home care) were eligible. Secretaries and pastors involved in carer support were decided to be included and were in this study referred to as HCPs. Informed, written consent was provided.

A study nurse employed at the Integrated Clinic, and the main author, identified candidates to approach for participation. Eligible HCPs were invited to participate by the study nurse, either by telephone or face-to-face. Practical considerations guided the composition of the groups. Groups consisting of the same profession were sought where possible. In cases where this was not possible, HCPs who worked together were gathered, independently of profession.

### Data collection

The participants’ age, profession, competence, and duration of work experience were collected by a written form. The focus groups were conducted by the main author (IR) assisted by the study nurse, and took place in private rooms at the participants’ working place. Based on existing literature [26–28], an interview guide was developed by all co-authors. The guide was used to ensure that all themes were covered. The questions and sub-questions were used actively if any of the topics were not covered in the group discussion [22]. The main interview topics were presented in Table 1. The interviewer (IR) ensured that all participants contributed in the discussion, encouraging the participants to comment on the others’ contributions, and allowing the participants to develop narratives and perspectives as freely as possible.

**Table 1:** Main interview topics

1 Extent of involvement in carer support	
2 Experiences with carer support	
3 Opinions on best possible carer support	
4 Experiences with carers’ support needs in different phases of the disease trajectory	
5 Opinions on and experiences with The Carer Support Needs Assessment Tool (CSNAT)	
6 Factors hampering provision of best possible carer support	
7 Factors facilitating provision of best possible carer support	
8 Education in carer support	

The Carer Support Needs Assessment Tool (CSNAT) is a 15-item self-report tool developed and validated in England [15], and translated into Norwegian. The tool forms part of a HCP-initiated, carer-led approach for carer assessment and support: carers’ completion of the tool is followed by a consultation with a nurse, allowing carers to discuss their support needs and priorities, and to further explain what supportive input would help [13]. CSNAT was recommended for use in the Orkdal region as part of the standardized care pathway. To explore if the HCPs had used CSNAT, and to explore their opinions on carers’ needs assessment, the interview guide included questions about CSNAT. Before asking about the use of CSNAT, the participants were given time to read through the tool.

### Data management and analysis

The interviews were audio-recorded and transcribed verbatim. Systematic text condensation was used for analyzes in a four step process according to Malterud [29]. Firstly, main themes were identified after the initial reading of the interviews. Secondly, meaning units, i.e. passages in the text containing information relevant to the research questions, were identified. Thirdly, the content of the meaning units of all interviews were condensed and grouped together. Fourthly, a summary of the main findings was done. All interviews were read and analyzed by two authors. The main author read and analyzed all; AKK, HSH and KS read two each. Verbatim quotes were presented for some of the results, not as evidence for all findings, but as illustration and explanation, with the aim to deepen understanding [30].

## Results

**Fig. 1:**
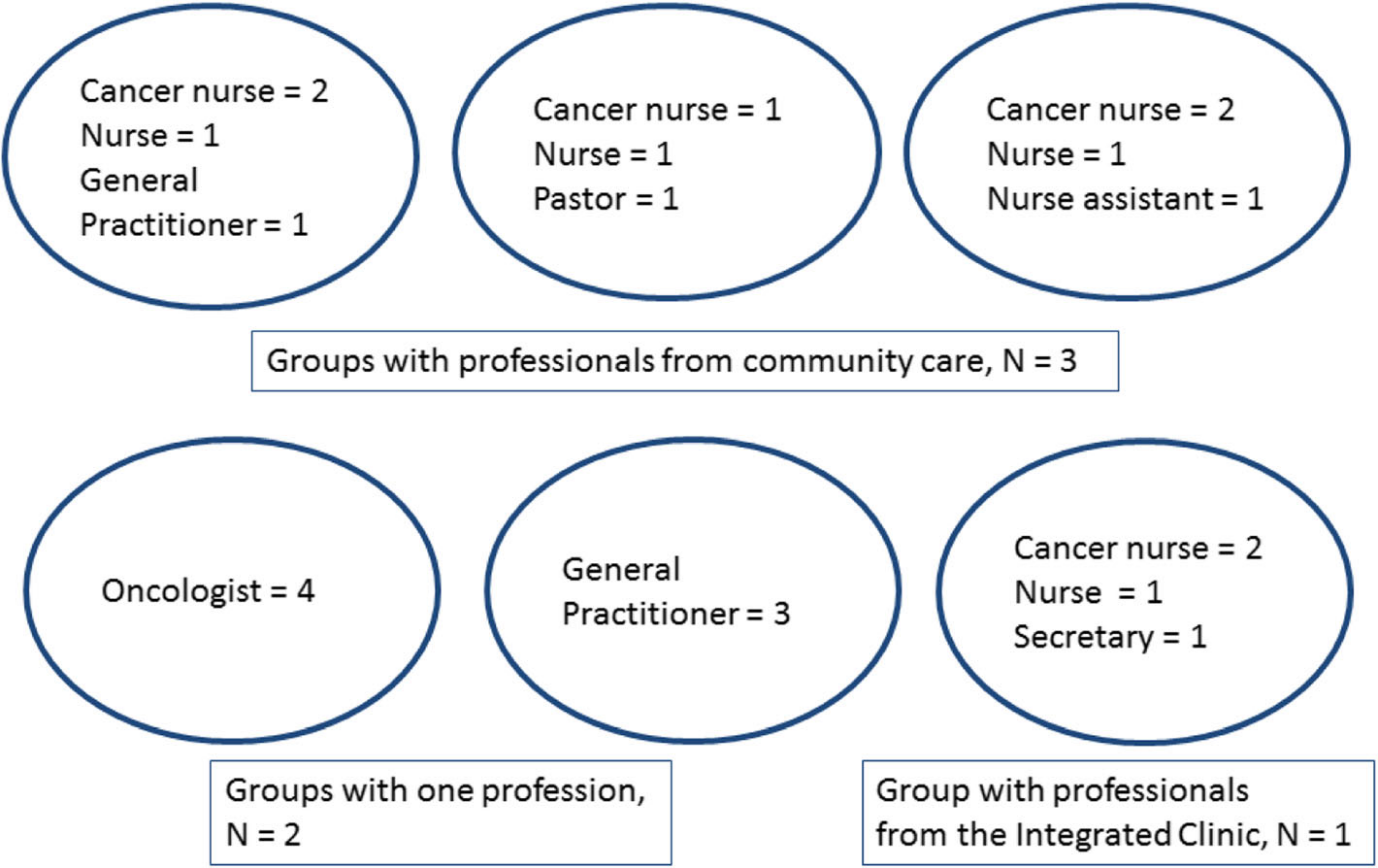
Composition of groups (*n* = 6)

Six focus group interviews with in total 21 health care professionals (HCPs) were performed. All HCPs approached agreed to participate; their characteristics were shown in Table 2. The planned number of four to five participants in each group was reduced to three to four for practical reasons. The composition of groups was presented in Fig. 1. The group interviews were on average 90 min long. The interviews took place between June 21st 2016 and March 30th 2017. At four groups, new information still emerged. After analyzes of six focus groups interviews, the authors agreed that saturation was reached.

**Table 2:** Characteristics of health care professionals participating in the focus groups (*n* = 21)

	N	Mean	Range
Gender			
Women	16		
Men	5		
Age		49	30–65
Years of work experience		20	5–38
Profession			
General Practitioner (GP)	4		
Oncologist	4		
Nurse	4		
Cancer nurse	6		
Pastor	1		
Secretary	1		
Nurse assistant	1		
Place of work - some had more than one			
Integrated clinic	5		
Palliative care unit local hospital	5		
Palliative care unit university hospital	4		
General Practitioners’ office	4		
Nursing home	8		
Home care	4

The findings in this study are based upon the study participants’ own perceptions and subjective experiences, analyzed through systematic text condensation [29]. In the following, the results from the interviews will be presented separately for the three research questions addressing: 1. how do HCPs support carers, 2. how do HCPs assess carers’ needs, and 3. what hampers HCPs’ carer support? The identified categories are listed in Table 3, and presented with corresponding numbers in the following. Quotes were presented to illustrate some of the findings.

**Table 3:** Main categories identified in the interview analyses

How do the health care professionals support carers?	
1	Dependent on profession, role, and context
2	Personal relationship
3	Personal skills and competence
4	Adjusted to the stage of the disease
How do health care professionals assess carers’ needs?	
5	Informal assessment of carers’ needs
What hampers health care professionals’ carer support?	
6	Lack of education
7	Lack systems for consultations
8	Lack of systems for documentation
9	Lack of systems for involving GPs
10	Lack of systematic spiritual care

### How do the health care professionals support carers?

1) (Corresponding numbers in Table 3). Health care professionals’ opportunities to support carers, and the way the support was provided, was dependent on the HCP’s profession, role, and context. Oncologists often informed and communicated with patients and carers together, since carers often accompanied the patients to consultations. The nurses, cancer nurses, and the nurse assistant had considerably more contact with the carers than the oncologists. They also provided a greater volume of carer support, and different support, e.g. they often listened to carers’ narratives about their situation and lives. The secretary met all patients and carers, and coordinated patient appointments to facilitate carer participation. The secretary was available for supportive talks with carers, but rarely at a deeper personal level, due to being located in a public area. The pastor came when called by the staff or by the carer, and talked with carers and patients, alone or together. Cancer nurses communicated with the whole family, especially at turning points in the disease trajectory, and coordinated care. Oncologists and general practitioners (GPs) not having medical responsibility for the carers, rarely talked with carers alone. The carer’s GP had the opportunity to talk with the carer alone, and with the patient and carer together, as expressed by a GP:

*GP: If I’m the carer’s GP, but not the patient’s, we talk a lot about it [the carer role]. Obviously, if I’m the GP of both, it’s easier to involve the two of them than if I’m not the carer’s GP. For you [nurse in home care], it‘s perhaps easier to gather both, since they are at home?*

2) Health care professionals reported that they aimed at establishing a personal relationship with the patient and the carer as soon as possible. The building of a personal relationship started as soon as the HCP met the carer for the first time. The first meeting took place at different stages of the patient’s disease trajectory, depending mostly on the healthcare setting. Whenever the carer was present at treatment and patient consultations, HCPs said they seized the occasion to pay attention also to the carers, asked about their wellbeing, and showed interest in their burdens and worries as carers.

*Cancer nurse: The primary thing is to get to know each other. At the first meeting, that they are being seen and heard, that we greet them, talk with them, show them that we are her for them. That’s extremely important for a start. We get to know each other, and then we’ll see what kind of needs that arise.*

Health care professionals said they received carers like guests, offering them coffee, and giving them their full attention.

*Nurse: I’ve always found it important to approach carers when they come, offer them coffee, and ask if there is anything I can do for them (…). I think it’s important to meet them, listen to them, hear what they have to say.*

Health care professionals reported that involving carers early facilitated their carer support later on. Not knowing the family, was reported to complicate carer support.

*Nurse: I was kind of thrown into a family, suddenly (…). It was a question of days (…). The patient was to die at home (…). No, that was hard. Really! I’ve had two cases like that, where we were involved all too late (…). And it was so difficult!*

3) Health care professionals reported that they involved themselves personally, and regarded personal skills and competence as important for their support. Health care professionals said that personal life experience helped them understand the carers’ situation. Experience with caring for, and losing a close one gave them a competence that could not be acquired through professional studies alone.

*Assistant nurse: In the school of life, we’ve been carers ourselves.*

Health care professionals reported that intuition helped them understand how to support the carers.

*Assistant nurse: Once in the room, you feel how you should proceed.*

Health care professionals said they made themselves available for dialogue with carers in case they wanted to talk, and sometimes encouraged them to ask questions.

*Cancer nurse: [I support carers] through dialogue. I don’t do much apart from that. Asking if they have questions, and if they want to talk.*

Health care professionals reported that the courage to address death was a personal competence needed in carer support. When HCPs addressed death, it helped carers and patients to talk about, and prepare for, the dying phase.

*General practitioner: There are huge differences as to how ready families are to talk about incurable disease and death. I often feel I need to tear down some walls by using words they avoid. To initiate such a dialogue is often very useful.*

4) Health care professionals said they adjusted their carer support to the stage of the patient’s disease. At diagnosis, the personal relationship was in focus, as reported above. When the treatment intention changed and/or when tumor directed treatment was ended, HCPs said they intensified their communication with, and counseling of the carers. Health care professionals reported that carers’ need for talks increased at these stages, sometimes due to changes in the patient’s behaviour. The patients could “change personality”, and criticize the carers. The oncologists used to have a consultation with the patient together with the carer. A nurse usually participated, also being available after the consultation.

Cancer nurse: The transition phase to only palliative treatment – to my experience, the support needs at that time are often greater than in the last phase.

At transition to community care, HCPs, including community cancer nurses, paid home visits to assess carers’ needs at this point, to inform about support available, and to make a support plan. Contact information to HCPs available at all times was given or updated.

*Cancer nurse: When I’m informed about a case by the hospital, the GP or the carer, I make contact, and certainly make a home visit to the patient (…). They receive information about the further plan, about what help and support we can offer.*

When the patient was dying, HCPs said that their personal presence and dedication became even more important. Community HCPs said they increased their availability and efforts to support the family. Nurses and GPs broke rules they normally followed, e.g. not handing out their private telephone number to a patient or carer. Furthermore, nurses, and sometimes GPs, called or visited carers at home, also outside their working hours.

*Cancer nurse: Then I feel you have to ignite another motor that lies deeper in here [points to the chest]. (…) The entire person has to be involved. When you know that somebody lives and dies at that very moment, (…) I’m prepared to make an extra effort. I feel that’s important.*

### How do the health care professionals assess carers’ needs?

5) Health care professionals reported that assessment of carers’ needs was done informally. Health care professionals said they tried to give unique support to each carer.

*Nurse: I don’t think there is a recipe for what to do when patients are seriously ill. It’s about getting to know them and being open to what’s important to them.*

Assessment of carers’ support needs was not systematized. Intuition was reported to be used to sense what carers and families needed.

*Cancer nurse: You kind of sense the atmosphere when you enter a room or a house, or open the door. It’s weird, and hard to explain, but you feel the atmosphere and how you should act: ‘Here I may need to be careful’.*

The Carer Support Need Assessment Tool (CSNAT) had only been used by HCPs at the Integrated Clinic, where one had used it several times, and three had used CSNAT once or twice each to gain practical experience. Health care professionals agreed that using CSNAT would help carers become aware of important needs that they would otherwise not have realized.

*Nurse: It’s easier to have a tool like that, instead of something based on your own thoughts. Here we have a list of issues they have to reflect upon, and the carers can address issues they might not have thought of themselves. So I think this can be of great help to us.*

Pros and cons for the use of CSNAT were reported. Health care professionals who had used CSNAT, said that the tool opened up for communication about important topics, helped structuring the carer consultations, and improved the collaboration with the family. Most HCPs found it difficult, or even unrealistic to implement another tool in clinical practice due to limited resources. Some said they feared that CSNAT would create expectations HCPs could not meet, and some felt that they already talked about the CSNAT items with carers. It was suggested that district cancer nurses should use CSNAT, especially when the patient was transferred to community care. GPs suggested that carers bring CSNAT completed to consultations with them, but said time, waiting room location, and current routines made it impossible to give CSNAT to carers to fill in when they came to the consultation.

*General practitioner: We do already touch these things, but it could absolutely be a tool to be used (…). We need help from others to be able to use such tools, because it’s almost hopeless to implement more forms, to be honest.*

### What hampers health care professionals’ carer support?

Five system deficiencies emerged as the answer to the third research question: “What hampers health care professionals’ carer support?” 6) A lack of education in carer support was reported. None of the professions reported that carer support was part of their basic formal education. The oncologists in particular underlined their lack of education. Oncologists were often in very demanding situations, e.g. communicating with whole families at critical moments when the patient’s disease had changed to the worse. They reported insufficient competence to handle these challenging communication situations. The oncologists described a great potential for improvement of their communication skills with carers and families.

*Oncologist: We handle pretty advanced, really, group discussions. And we really don’t have much background for it, do we?*

*Oncologist: And I think that there’s an enormous potential, really, in developing this (…). Our ability to communicate can be improved by some simple measures,- and it’s needed!*

Carer support was, however, reported to be part of specialization or further education by all professions. GPs and nurses said that the education provided as part of the Orkdal Model increased their competence. Cancer nurses learnt about carer support, including how to support carers and families, through their speciality, and additionally through training particularly for cancer nurses and pastors in the Orkdal region. Community cancer nurses with such competence were central in the organization of support for patient and carer, coordinated the palliative care team, and taught other members how to support carers.

*Nurse: I see a great difference after she started here as a cancer nurse, she’s got another way of thinking (…), it’s more systematic now (…). We learn a lot from her.*

7) Health care professionals reported that they wanted carers to take part in patient consultations from the beginning, but that a system for consultations with carers was lacking. Health care professionals reported that standard invitation to all carers to accompany the patient to treatment and consultations was not allowed; asking for patient consent was needed in each case. Health care professionals said they tried to remember to encourage patients and carers to come together to patient consultations. Health care professionals said that if carers did not accompany the patients to consultations, they risked extra work, and carers risked being less informed and prepared than the patients, with negative consequences for carer support throughout the disease trajectory.

*Oncologist: But we also try to be extremely conscious to always encourage the patient to bring the carer [to consultations], and we really prefer that they do. If carers haven’t been offered to join in when we have a talk with the patient, I simply have to do double work, ‘cause then carers come afterwards and want the same information. So it’s best to plan in advance to have a consultation with carer, patient, and doctor together.*

8) A lack of possibility for full documentation of carer support was reported. Health care professionals said they were supposed to document patient relevant information from carers in the patient record. However, the patients had the right to read their record. HCP reported that information regarding carers relevant for both patient treatment and carer support could not be documented in the patient record, thus hampering the transfer of information between HCPs and levels of care.

*Oncologist: There’s no place to document what we have talked about with carers, and what we feel are problems carers have (…). It’s not right to document it in the patient record.*

*GP: I know that a lot of good dialogues with carers take place at the Integrated Clinic, but we don’t find it in the discharge summary. So the GP usually knows nothing about the carers, really, I think.*

9) A lack of systems for involving carers’ GP was reported. GPs said that they too often were not involved in the care of patient and carer when the patient with advanced cancer received specialist health care. Often, the patient received all follow-up in specialist health care. GPs wanted the specialists to encourage patients and carers to be in contact with the GP throughout the disease trajectory. Despite heavy work load, they were ready to do more for patients with advanced cancer and their carers. GPs suggested that specialist health care could organize a meeting with the GP at transfer to community care when tumor directed treatment was terminated. GPs were of the opinion that their involvement at the end-of-life could prevent overtreatment and unnecessary hospitalizations. Cancer nurses reported that GPs’ involvement the last days of life made the family feel safe. The cancer nurses asked GPs to do home visits when needed. GPs appreciated this coordination done by cancer nurses. However, finding time for home visits was not easy.

*General practitioner: The challenge is often to get the patient back from the hospital. I find it very meaningful to contribute to a good last phase (…). I think it would be ideal to have a meeting with patients and carers before it becomes hyper acute.*

10) A lack of systematic spiritual care was reported. Health care professionals found spiritual care important for patients’ and carers’ peace and wellbeing. Despite this, they reported a lack of own spiritual competence, a lack of system for assessment of spiritual needs, and a lack of system for involvement of professional spiritual care. The examples of spiritual care HCPs mentioned were mainly pastoral care. Health care professionals said that they could sing for or pray with the patient, regardless of being believers themselves. Some HCPs asked carers if they wanted them to call a pastor when the patient was dying, but said they sometimes forgot to offer pastoral care, or involved a pastor too late.

*Cancer nurse: We may need to do more when it comes to calling a pastor in time (…). The pastor may become a partner to communicate with (…). It doesn’t need to be that spiritual, but just talking about certain topics.*

## Discussion

This qualitative study aimed, through focus group interviews, to explore and describe how health care professionals (HCPs) in a rural district of Mid-Norway supported carers of patients with advanced cancer, how carers’ needs were assessed, and what hampered carer support. Through systematic text condensation [29], ten categories were identified. In summary, these covered education, the personal relationship between HCPs and carers, and lack of organizational structures for carer support.

Lack of education in carer support was reported by all professions in the focus groups. Palliative care includes physical, psychological, social, and spiritual care, and is hence to be delivered by a multidisciplinary team [2]. All professions in the team need palliative care competence, and building of competence is a national priority [16]. A recent Lancet Oncology Commission Commission on cancer palliative care described a lack of basic and specialist palliative care competence at all levels of health care [7]. The authors stated that there is much less focus on training in psychological and spiritual care than physical care training, and highlighted the need for education of all professions [7]. In the present study, oncologists reported a lack of education in communication with carers. Physicians’ need for communication training has been addressed also elsewhere [31]. The importance of a personal relationship between carers and HCPs reported in this study has been supported by a recent study in carers [28], and underlined in the Lancet Oncology Commission report [7]. Recommendations from the American Society of Clinical Oncology (ASCO) include building of relationship with patients and carers [32]. Psychologists have regarded the quality of the therapeutic relationship as the most important factor for successful psychotherapeutic interventions [33]. Carers of patients with advanced cancer have reported that not receiving personal attention and care from HCPs complicated their grief, e.g. creating feelings of anger and of being abandoned [27]. Such a personal relationship between HCPs and carers early in the disease trajectory may be facilitated by achieving integration of oncology and palliative care, an essential element of the cancer palliative care offered in the Orkdal region. Early integration of oncology and palliative care has been reported to have positive effects for both patients and their families, and is recommended [7, 32, 34].

The findings of the present study revealed a lack of organizational structures for carer support. An overall hampering factor for good carer support was that the health care system was described as being mainly designed for the patients, and not for the carers. The World Health Organization (WHO) definition described palliative care for families as a support system to enhance their coping [2]. Carers have reported lack of systematic care to cause distress [35]. A study from Finland found that carers’ needs for information and emotional support in oncology were mostly unmet by HCPs, and that carers may easily be forgotten [36]. The lack of system for systematic carer support was reported to cause extra work. E.g. the individual HCP had to ask each patient to bring the carers to consultations, thus entrusting care for carers to individual initiative. However, patients’ right to confidentiality is a considerable barrier to the implementation of systematic invitation to patient consultations and to systematic information to carers. Furthermore, the extent of communication between carers and HCPs, and between carers and patients, depends on how much communication the patients want [37]. Carers receiving insufficient information about diagnosis and death due to the patient wanting less information, have reported that the lack of information led to negative consequences for their relationship to the patient, for their preparedness for death [28], and for the bereavement [27]. Information about diagnosis and dying is by HCPs in the present and in a previous study [38] regarded as helpful for carers’ coping.

An overview of carer support needs through the cancer trajectory stated that HCPs tend to provide support based on their impressions, and not on systematic assessment directly with the carers [39]. The present study confirmed this as use of personal skills, including intuition, was reported to be important in carer support. HCPs’ unstructured assessment of carers’ needs reported in this study, was also found in a recent study on the role of the Carer Support Needs Assessment Tool (CSNAT) in home care [13]. The same study found that HCPs were surprised by the carers’ answers when actually assessing the carers’ support needs [13]. Carers rarely ask, and have reported that they do not know what to ask about, or what support they may need to ask for, e.g. at discharge from hospital, but need concrete questions from HCPs like those in CSNAT [40]. Implementation of CSNAT has been shown to reduce carer burden during caregiving [41], improve carers’ psychological and physical health, make fulfillment of patients’ wish to die at home more probable, and improve grief after bereavement [42]. Use of CSNAT is a professional way of assessing carer needs that allows the carers to define their needs and what support they prefer, thus empowering the carers, and involving them in decision processes [43]. Personal skills including intuition, and systematic assessment should not be viewed as conflicting, but as complementary tools to improve care. This study suggests that use of CSNAT at transition from specialist to community care could be helpful, and contribute to a seamless transition of patients and carers between places and levels of care.

GPs may have a valuable knowledge about the patient and their family, acquired through years of follow-up. Collaboration between specialists and GPs has been recommended [2]. However, GPs in this study said they often lost contact with patient and family during follow-up in specialist health care. Insufficient information to GPs from specialist care at patient transfer to community care has been reported [44]. Collaboration between oncologists and GPs in palliative care improves patients’ health outcomes, and there is reason to believe that carers also benefit from such a collaboration [45]. GPs in the present study suggested that a meeting with the carer, the patient, the patient’s GP, and staff be arranged at the hospital before transition from specialist to community care. Specialists were encouraged to motivate patients and carers to visit their respective GP during the cancer disease trajectory.

Health care professionals are supposed to document all information relevant for patient treatment and care. However, the patients’ right to read the patient record would require carers’ consent to document sensitive carer related information, including information relevant for patient treatment and carer support. In a recent British qualitative study, carers reported an unmet need for exchange of carer-information between HCPs in specialist to community care, and carers’ expectations for such information transfer to take place were low [46]. As long as the patient record remains the only place for documentation of carer support, HCPs could make it a routine to ask for carer consent to document in the patient record. However, in order to make systematic carer support possible, a separate carer record was recommended in a recent Hospice UK report [47], a recommendation supported by the present study.

### Strengths and limitations

A strength of the study was that all HCPs consented to participate. The response rate probably reflected HCPs’ willingness to contribute to improve carer support within cancer care in the Orkdal region, and that competent and highly motivated HCPs were asked to participate. Another strength was that the HCP experiences explored, stemmed from various contexts across levels of care. A limitation was however that the study was confined to one geographical area, which may reduce the transferability to other settings. A further weakness was that the three professions secretary, pastor and nurse assistant were represented by only one each. This study has focused on a cancer population, but the findings may be relevant for HCPs’ carer support to carers of patient with incurable diseases other than cancer.

## Conclusion

Health care professionals (HCPs) in specialist and community cancer care used their personal competence to build a relationship to carers of patients with advanced cancer as the fundament for provision of carer support. Health care professionals described the carer support offered as tailored to the individual carer and to the phase of the patient’s disease.

However, educational deficiencies were reported. Furthermore, the quality of the carer support depended heavily on the individual HCP’s competence and engagement, as systems to ensure systematic carer support were not implemented. Carer support was hampered by systems in health care being tailored to the patients, and not to the carers. Assessment of carer needs was unstructured. It may be hypothesized that these results are generalizable to other regions and other areas of medicine.

Organizational changes to improve carer support are needed, and may include: 1) education in carer support integrated in all professions’ educational programs and further education, including communication with families, and spiritual care, 2) use of standardized care pathways, including systematic carer needs assessment, 3) systematic involvement of General Practitioners, and 4) a system for separate, comprehensive documentation of clinical work with carers.

## Data Availability

The audio-taped and transcribed interviews are not publicly available due to private details about health care professionals, patients, families and carers, but are available from the corresponding author on reasonable request.

## Abbreviations

CSNAT: The Carer Support Needs Assessment Tool
GP: General practitioner
HCP: Health care professional
RCT: Randomized controlled trial
